# Medical Statistics

**Published:** 1837-07

**Authors:** 


### MEDICAL STATISTICS.

### *Statistics of Suicide.*

[WE find, among the miscellanies of *Wildberg's Jahrbuch d. ges. S. A. für* 1837, several scattered notices on the statistics of suicide. We have collected them, and here present them at one view to our readers.]

*Paris.* The number of suicides in Paris were, for

| | | |
|---|---|---|
| 1830, 269 | 1832, 369 | 1834, 436 |
| 1831, 377 | 1833, 333 | 1835, 477. |

This is in the ratio of about *one suicide* out of every *one hundred deaths;* while, observes the reporter, in England, the land of suicide (!), the proportion is not greater than *one* out of every *five hundred deaths.*

[Although we think it cannot be denied that the average number of suicides, in relation to the total deaths, is considerably less in England than in France, we much doubt whether the ratio here given, of $\frac{1}{500}$, be correct; since so little attention is paid by the government of this country to the collection and publication of the details.]

*Naples.* During the year 1836, out of a population of 357,283 souls, there were not more than thirty-one cases of suicide.

*St. Petersburg.* The number of suicides in this capital, for the years 1831, 1832, and 1833, did not exceed 104; which presents an average of about thirty-four annually. They were mostly confined to the lower classes of society.

*Bohemia.* In this kingdom, during the year 1835, the deaths amounted to 119,438. Of these there were 188 cases of suicide, which is in the ratio of about one suicide out of six hundred deaths.

*Lower Austria.* From the 1st November, 1835, to the end of October, 1836, there were 49,556 deaths, and out of these 109 cases of suicide. This gives a ratio of about one in 457.

*Wildberg's Jahrbuch d. ges. S. A.* 3 B. 2 h. 1837.

---

### *Statistics of Stillborn Children.*

[WE consider this subject important in relation to infanticide. When, in a doubtful case, a medical jurist attempts to estimate the probability of a child having come into the world living or dead, he assuredly ought to be prepared with a knowledge of the results which have been derived from statistical enquiries relative to live and still birth. If this kind of knowledge answer no other purpose, it must have the effect of rendering him cautious in the expression of an opinion. The subject, however, is not without interest to the general practitioner in numerous other points of view. In the following extracts we shall not confine ourselves strictly to foreign sources, being anxious to render our view of the subject as complete as we can.]

I.

*Bohemia.* In 1835, the births were in this kingdom 160,871, of which 2,561 children were stillborn. This nearly gives a ratio of only one child born dead out of every sixty-three births; an extraordinarily low proportion.

*Lower Austria.* From 1st November, 1835, to the end of October, 1836, the births were 49,658; of which number, 1,215 were stillborn children. This is nearly in a ratio of one in forty-one. The still-births were twice as numerous among *legitimate* as among *illegitimate* children; and, with regard to *sex*, the stillborn males were to females as about four to three among the legitimate births. Among the stillborn illegitimate children, the sexes were nearly equal.

*Pressburg.* In the year 1835, there were born 1,385 children; of which, fifty-five were born dead. This is a ratio of about one in twenty-five.

*Innsbrück.* From the 1st November, 1834, to the end of October, 1835, the births in this town amounted to 314, of which seventeen were stillborn; a ratio of one in eighteen. The stillborn males, even in this small number, were to the females as two to one. *Wildberg's Jahrbuch, loc. cit.*

II.

*Geneva (City).* During the ten years from 1814 to 1823, there were born, as a mean annual average, 511 children; out of which number thirty-four were born dead, giving a ratio of one to fifteen for the still-births. In the ten years from 1824 to 1833, there were born, as an annual average, 581 children; of which thirty were born dead, about one in nineteen. The mean annual births were, however, for the whole period of *twenty years* over which the observations extend, 546; and the mean annual number of still-births was, for the same period, thirty-two, about one in seventeen. Of the mean annual births, the males were to the females as 284 to 262; in the still-births, as eighteen to fourteen. We learn by this that, as the preponderance of total births is on the side of the male sex, so is the preponderance of still births. It is to be observed, however, that the number of children born dead was liable to great yearly fluctuations, being sometimes as high as one-eleventh, and sometimes as low as one twenty-seventh of the total births.

In a medico-legal view, it is interesting to ascertain the ratios of the stillborn among *legitimate* and *illegitimate* children, whenever this is practicable; since the great majority of alleged cases of child-murder lie among the *illegitimate*.

Out of 9,833 *legitimate* births, during the whole period of twenty years, 517 children were born dead, or one child was born dead in every nineteen legitimate births. Out of 1,092 *illegitimate*, there were 129 born dead, or one in 8.4 cases.

These results, deduced from observations on nearly eleven thousand births, show that the stillborn are more than *twice as numerous* among illegitimate as among legitimate children This is exactly what we might expect; since the circumstances under which illegitimate children are born tend to render their coming into the world alive very uncertain. The want of care and attention at the time of delivery, which generally takes place in secret, as well as the anxiety of mind under which the mother is commonly labouring, are facts that perhaps sufficiently explain the difference observed. The result of the Genevan observations is, it will be seen, entirely opposed to that deduced from the statistical survey of Lower Austria. Notwithstanding that, in the two cases, the total births of the illegitimate children are nearly *equal;* and that, in respect to legitimate children, the total births in Lower Austria are *five times as numerous* as those of Geneva; and, therefore, admitting only one-fifth of the possible error which would attach to those of Geneva, we are inclined to place greater confidence in the Genevan result, not only from the very high probability which attends it, but also from the circumstance that observations extending over a long series of years are much more to be trusted than those which are confined to a single year.

Among the illegitimate, it was found that there was a slight preponderance of *male* over female births, as well among those children which were born living as among those which were born dead. The number of stillborn *males*, taking the total births, was much greater than that of females. The ratio for the whole period of the Genevan tables, twenty years, was as four to three. This difference has been ascribed, and perhaps with justice, to the generally greater size of the head and body in the male; conditions which necessarily expose the child to greater risk during delivery. As a summary of the stillborn to the total births, the following statement may be taken:

| | |
|---|---|
| 1 *male* born dead in . . . | 15.59 births. |
| 1 *female* . . . . . | 18.60 — |
| 1 child . . . . . . | 16.91 — |

*Paris.* To his remarks on Geneva, the author of the report, M. Mallet, has appended a statistical summary derived from observations made during a period of *thirteen years*, namely, from 1819 to 1832, in the capital of France. It is as follows:

| | |
|---|---|
| 1 *male* was born dead in . . | 16.48 births. |
| 1 *female* . . . . . | 19.67 — |
| 1 child . . . . . | 17.90 — |

*Marseilles.* According to the statistical survey of the department of the Bouches du Rhone, in the city of Marseilles there is one child born dead in sixteen births. *Ann. d'Hygiène et de Méd. Lég. Janvier*, 1837.

III.

*Prussia.* In the recently published volume of the Statistical Society of London, we have met with a valuable paper founded on the researches of M. Hoffmann, of Berlin. It represents a statistical view of the births and deaths in the Prussian states during a period of *fifteen years*, from 1820 to 1834. It is entitled to our consideration, not only from the reputation of the compiler, but from the fact of its comprising several *millions* of births, extending over a long period of time. We shall confine our extracts to that portion of the paper which is relevant to the question now under examination.

| | |
|---|---|
| In the fifteen years, there were born in the Prussian states, | 7,593,017 |
| Of these, there were born dead, . . . . . . | 257,068 |

This gives a ratio of rather above one child born dead in every 29.14 births; that is to say, the stillborn formed rather more than 3.38 per cent. of the total births.

This vast compilation also shows the same striking difference in the mortality of male and female children during birth which we have already had occasion to point out. M. Hoffmann refers this difference to the same causes as ourselves. In the above period of fifteen years there were born, of *males* 3,906,544, and of *females* 3,686,473; of which were born dead, *males* 147,705, and of *females* 109,363. These numbers give a general ratio of males born dead to females of 1.35 to one; or, for every one hundred stillborn *females* there were 135 stillborn *males.*

The influence of legitimacy and illegitimacy is not noticed in this paper, probably from the great difficulties which must have attended the collecting of satisfactory evidence on this point, among so many millions of births.

*Trans. Stat. Soc. Lond.* 1837. Vol. i. Part 1., p. 132.

IV.

From October, 1835, to October, 1836, in the Lying-in Charity of Guy's Hospital, there were 630 births, and, among these, thirty-four children were born dead, in about *equal proportions* of the two sexes. It is worthy of remark, that only *eight* out of the total number of deliveries were premature; an important circumstance, and one which ought always to be ascertained, to allow of fair statistical comparisons; since it is beyond all question that the ratio of live to still births must vary for the different periods of gestation at which a child is born. The ratio of the still-born here amounts to one-eighteenth of the total births.

Out of 766 applicants to the Charity during the year, it was ascertained that

643 had already given birth to 2,460 children; of which, 133 were stillborn,—a ratio of one in eighteen. It is curious thus to find that the ratios should be alike, although, as will be perceived, the births from which they were deduced were four times as numerous in the one case as in the other. This fact, in our view, furnishes at the same time strong intrinsic evidence of our having reached a near approximation to the truth.

*Dr. Ashwell, in Guy's Hospital Reports;* IV. *April,* 1837.

[Our readers will have perceived that some of the results differ very widely; but, if we except Bohemia ($\frac{1}{63}$), and Lower Austria ($\frac{1}{41}$), which numbers representing, as they do, merely *one annual result,* and differing so materially from all the others in our list, we do not hesitate to exclude from our calculation, we shall have a mean result of $\frac{1}{20}$: i. e. that *one child out of twenty* is stillborn. This result, it is to be observed, is deduced, at the lowest computation, from nearly *eight millions* of births.]

